# The genome sequence of the short-fringed mining bee, *Andrena dorsata *(Kirby, 1802)

**DOI:** 10.12688/wellcomeopenres.19756.1

**Published:** 2023-08-30

**Authors:** Steven Falk, John F. Mulley, University of Oxford and Wytham Woods Genome Acquisition Lab, Darwin Tree of Life Barcoding collective, Wellcome Sanger Institute Tree of Life programme, Wellcome Sanger Institute Scientific Operations: DNA Pipelines collective, Tree of Life Core Informatics collective, Darwin Tree of Life Consortium

**Affiliations:** 1 Independent researcher, Kenilworth, England, UK; 2 Bangor University, Bangor, Wales, UK

**Keywords:** Andrena dorsata, short-fringed mining bee, genome sequence, chromosomal, Hymenoptera

## Abstract

We present a genome assembly from an individual female *Andrena dorsata* (the short-fringed mining bee; Arthropoda; Insecta; Hymenoptera; Andrenidae). The genome sequence is 277.3 megabases in span. Most of the assembly is scaffolded into 3 chromosomal pseudomolecules. The mitochondrial genome has also been assembled and is 16.11 kilobases in length. Gene annotation of this assembly on Ensembl identified 10,916 protein coding genes.

## Species taxonomy

Eukaryota; Metazoa; Eumetazoa; Bilateria; Protostomia; Ecdysozoa; Panarthropoda; Arthropoda; Mandibulata; Pancrustacea; Hexapoda; Insecta; Dicondylia; Pterygota; Neoptera; Endopterygota; Hymenoptera; Apocrita; Aculeata; Apoidea; Anthophila; Andrenidae; Andreninae; *Andrena*; *Simandrena*; *Andrena dorsata* (Kirby, 1802) (NCBI:txid1411666).

## Background

The short-fringed mining bee ( *Andrena dorsata*) is a small (8–9 mm body length ( Warzecha *et al.*, 2016)) ground-nesting solitary bee found in western Europe that is especially common in south-eastern England, with scattered records further north and in south and north Wales ( NBN Atlas Partnership, 2021). As with other mining bees, females dig a nest burrow and provision each egg with pollen collected from nearby flowers. *A. dorsata* is a bivoltine species ( Warzecha *et al.*, 2016), with one generation in spring (peaking in April in the UK) and another in summer (peaking in July), and the more southerly distribution may reflect the need for warmer temperatures to facilitate production of two generations in a year. Whilst some members of the genus *Andrena* are communal, with many females nesting independently but in close proximity, *A. dorsata* is a true solitary bee ( Wcislo & Fewell, 2017). The conservation status of *A. dorsata* is currently unclear, as this species, like some 56.7% of all European bee species is classified as ‘Data deficient’ by the ICUN ( Nieto *et al.*, 2014; Potts *et al.*, 2016).

Karyotype information for bees is sparse, with only around 200 of the estimated 18,00–20,000 bee species having been investigated ( Cunha *et al.*, 2021), and members of the genus *Andrena* are at the low end of the bee chromosome scale, with a diploid chromosome number (2 *n*) of 6, with the full bee range spanning 6 to 58 chromosomes. This *A. dorsata* genome, together with those of other bee species, will be useful for the identification of the processes that have so radically altered chromosome number in bees, and for the characterisation of the genome-level processes underlying increased GC content in this and other members of the genus ( Sless *et al.*, 2022): the three chromosomes of *A. dorsata* range in GC content from 42.5–43%, whereas the honeybee ( *Apis mellifera*, 2 *n* = 32) genome average is 10% lower at 34% ( Wallberg *et al.*, 2019).

## Genome sequence report

**Figure 1.**
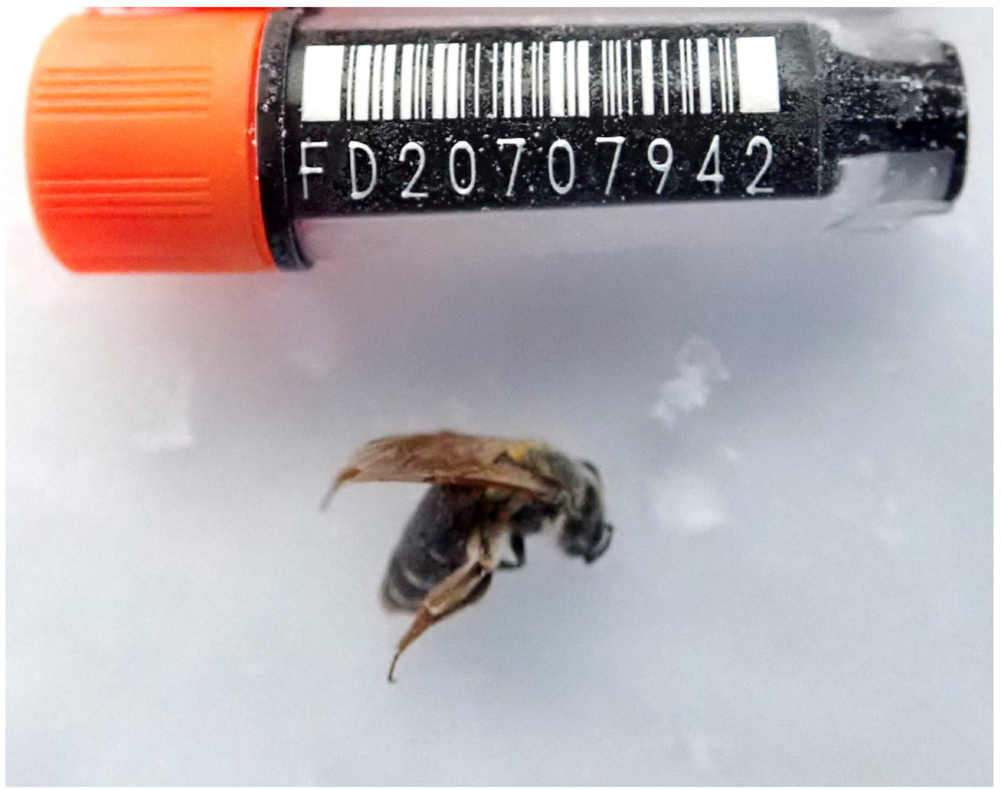
Photograph of the *Andrena dorsata* (iyAndDors1) specimen used for genome sequencing.

The genome was sequenced from one female *Andrena dorsata* ( Figure 1) collected from Wytham Woods, Oxfordshire, UK (51.77, –1.33). A total of 40-fold coverage in Pacific Biosciences single-molecule HiFi long reads and 85-fold coverage in 10X Genomics read clouds were generated. Primary assembly contigs were scaffolded with chromosome conformation Hi-C data. Manual assembly curation corrected 20 missing joins or mis-joins and removed 2 haplotypic duplications, reducing the assembly length by 1.27% and the scaffold number by 3.68%, and increasing the scaffold N50 by 2.3%.

**Figure 2.**
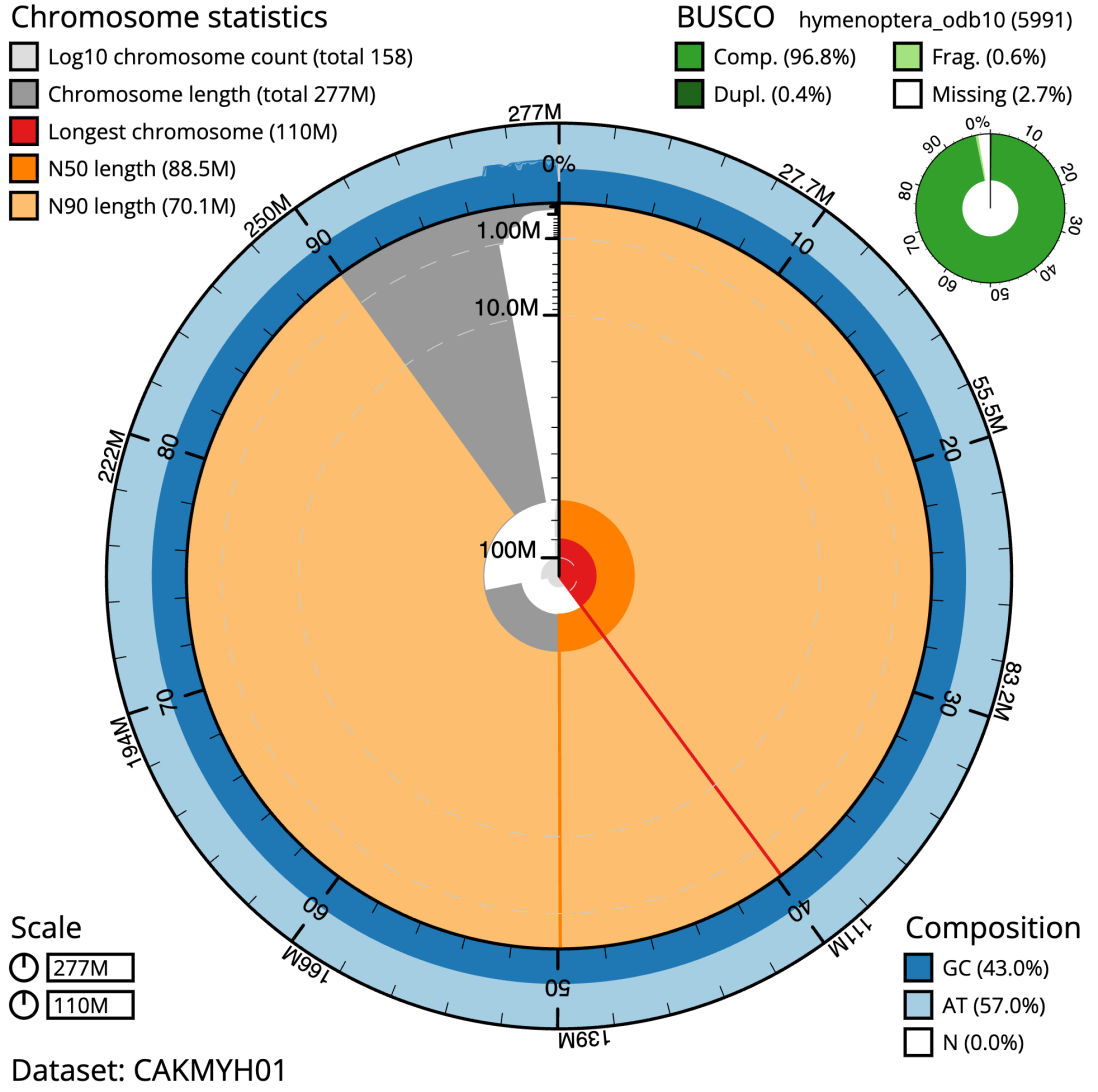
Genome assembly of *Andrena dorsata*, iyAndDors1.1: metrics. The BlobToolKit Snailplot shows N50 metrics and BUSCO gene completeness. The main plot is divided into 1,000 size-ordered bins around the circumference with each bin representing 0.1% of the 277,337,111 bp assembly. The distribution of scaffold lengths is shown in dark grey with the plot radius scaled to the longest scaffold present in the assembly (110,492,342 bp, shown in red). Orange and pale-orange arcs show the N50 and N90 scaffold lengths (88,484,888 and 70,131,001 bp), respectively. The pale grey spiral shows the cumulative scaffold count on a log scale with white scale lines showing successive orders of magnitude. The blue and pale-blue area around the outside of the plot shows the distribution of GC, AT and N percentages in the same bins as the inner plot. A summary of complete, fragmented, duplicated and missing BUSCO genes in the hymenoptera_odb10 set is shown in the top right. An interactive version of this figure is available at https://blobtoolkit.genomehubs.org/view/iyAndDors1.1/dataset/CAKMYH01/snail.

The final assembly has a total length of 277.3 Mb in 157 sequence scaffolds with a scaffold N50 of 88.5 Mb ( Table 1). Most (97.03%) of the assembly sequence was assigned to 3 chromosomal-level scaffolds. Chromosome-scale scaffolds confirmed by the Hi-C data are named in order of size ( Figure 2– Figure 5; Table 2). While not fully phased, the assembly deposited is of one haplotype. Contigs corresponding to the second haplotype have also been deposited. The mitochondrial genome was also assembled and can be found as a contig within the multifasta file of the genome submission.

The estimated Quality Value (QV) of the final assembly is 53.5 with *k*-mer completeness of 99.99%, and the assembly has a BUSCO v5.3.2 completeness of 96.8% (single = 96.4%, duplicated = 0.4%), using the hymenoptera_odb10 reference set ( *n* = 5,991).

**Table 1.** Genome data for *Andrena dorsata*, iyAndDors1.1.

Project accession data		
Assembly identifier	iyAndDors1.1	
Species	*Andrena dorsata*	
Specimen	iyAndDors1	
NCBI taxonomy ID	1411666	
BioProject	PRJEB48400	
BioSample ID	SAMEA7746464	
Isolate information	iyAndDors1, female; thorax (DNA sequencing), head (Hi-C scaffolding)	
Assembly metrics *		*Benchmark*
Consensus quality (QV)	53.5	*≥ 50*
*k*-mer completeness	99.99%	*≥ 95%*
BUSCO **	C:96.8%[S:96.4%,D:0.4%], F:0.6%,M:2.7%,n:5,991	*C ≥ 95%*
Percentage of assembly mapped to chromosomes	97.03%	*≥ 95%*
Sex chromosomes	-	*localised homologous pairs*
Organelles	Mitochondrial genome assembled	*complete single alleles*
Raw data accessions		
PacificBiosciences SEQUEL IIe	ERR7254653	
10X Genomics Illumina	ERR7220494, ERR7220495, ERR7220496, ERR7220493	
Hi-C Illumina	ERR7220497	
Genome assembly		
Assembly accession	GCA_929108735.1	
*Accession of alternate haplotype*	GCA_929114765.1	
Span (Mb)	277.3	
Number of contigs	182	
Contig N50 length (Mb)	30.1	
Number of scaffolds	157	
Scaffold N50 length (Mb)	88.5	
Longest scaffold (Mb)	110.5	
Genome annotation		
Number of protein-coding genes	10,916	
Number of non-coding genes	3,489	
Number of gene transcripts	24,323	

* Assembly metric benchmarks are adapted from column VGP-2020 of “Table 1: Proposed standards and metrics for defining genome assembly quality” from ( Rhie *et al.*, 2021). ** BUSCO scores based on the hymenoptera_odb10 BUSCO set using v5.3.2. C = complete [S = single copy, D = duplicated], F = fragmented, M = missing, n = number of orthologues in comparison. A full set of BUSCO scores is available at https://blobtoolkit.genomehubs.org/view/iyAndDors1.1/dataset/CAKMYH01/busco.

**Figure 3.**
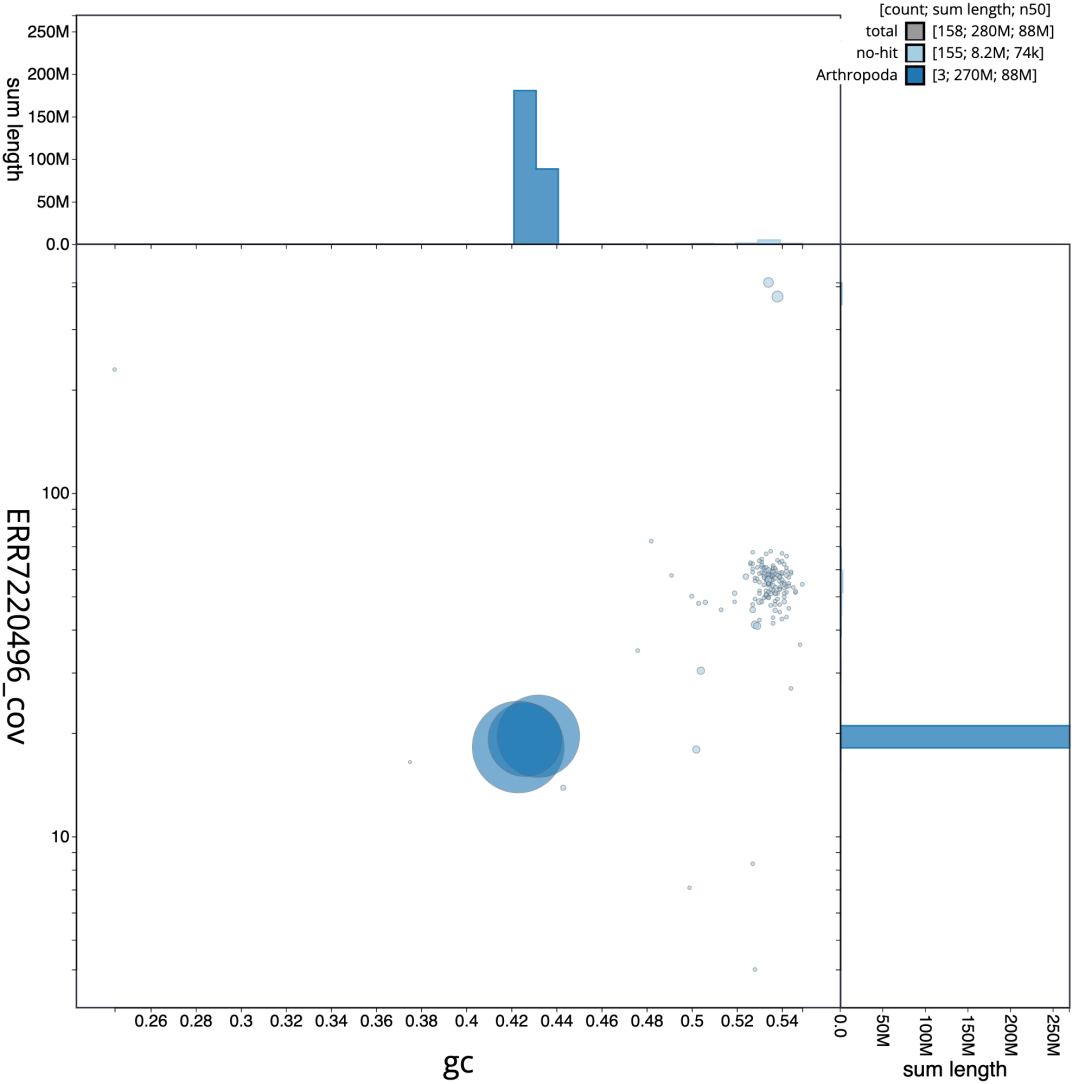
Genome assembly of *Andrena dorsata*, iyAndDors1.1: BlobToolKit GC-coverage plot. Scaffolds are coloured by phylum. Circles are sized in proportion to scaffold length. Histograms show the distribution of scaffold length sum along each axis. An interactive version of this figure is available at https://blobtoolkit.genomehubs.org/view/iyAndDors1.1/dataset/CAKMYH01/blob.

**Figure 4.**
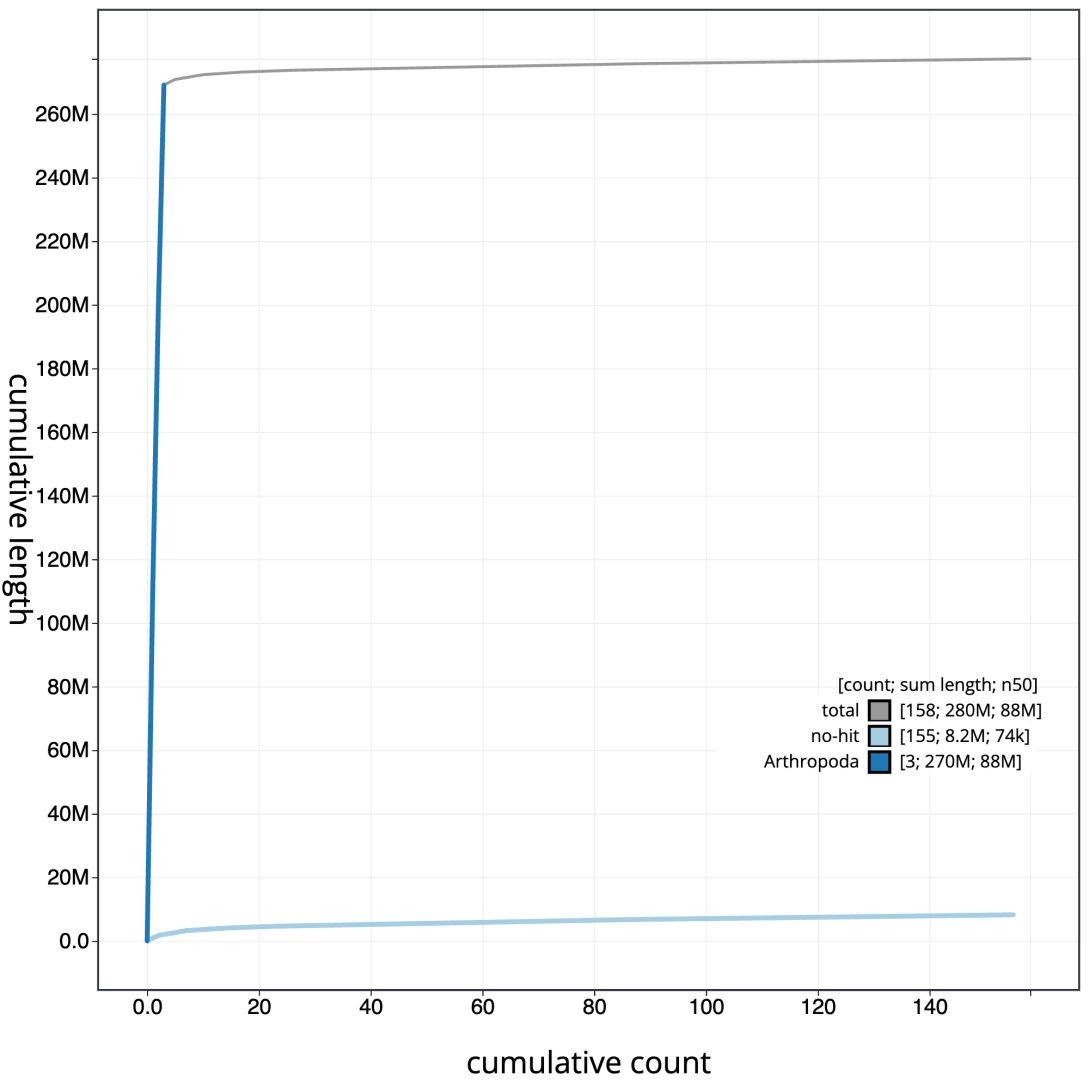
Genome assembly of *Andrena dorsata*, iyAndDors1.1: BlobToolKit cumulative sequence plot. The grey line shows cumulative length for all scaffolds. Coloured lines show cumulative lengths of scaffolds assigned to each phylum using the buscogenes taxrule. An interactive version of this figure is available at https://blobtoolkit.genomehubs.org/view/iyAndDors1.1/dataset/CAKMYH01/cumulative.

**Figure 5.**
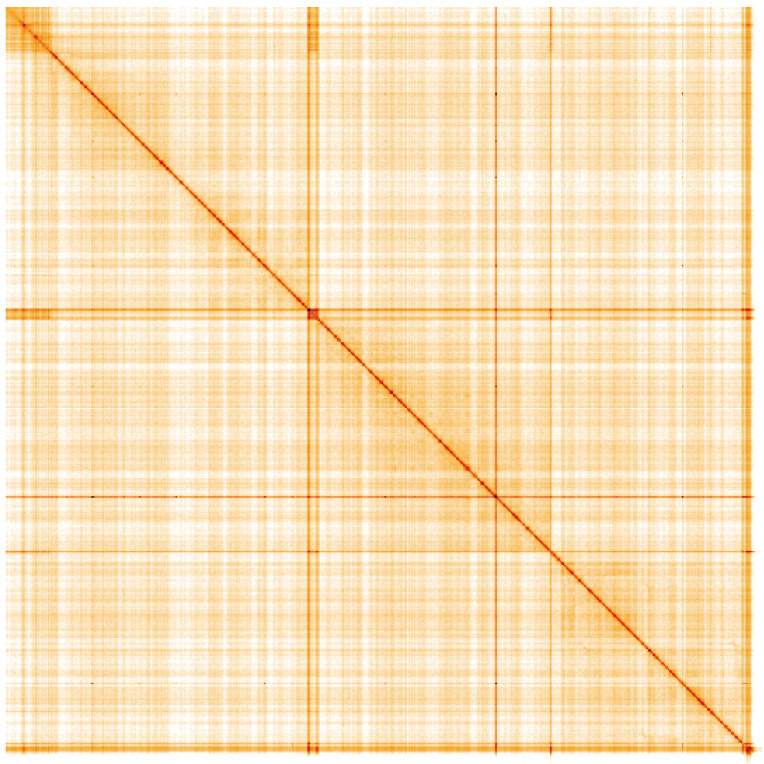
Genome assembly of *Andrena dorsata*, iyAndDors1.1: Hi-C contact map of the iyAndDors1.1 assembly, visualised using HiGlass. Chromosomes are shown in order of size from left to right and top to bottom. An interactive version of this figure may be viewed at https://genome-note-higlass.tol.sanger.ac.uk/l/?d=AD908eZZTU2AuDnxO0UBbA.

**Table 2.** Chromosomal pseudomolecules in the genome assembly of *Andrena dorsata*, iyAndDors1.

INSDC accession	Chromosome	Length (Mb)	GC%
OV815487.1	1	110.49	42.5
OV815488.1	2	88.48	43.0
OV815489.1	3	70.13	42.5
OV815490.1	MT	0.02	25.0

Metadata for specimens, spectral estimates, sequencing runs, contaminants and pre-curation assembly statistics can be found at https://links.tol.sanger.ac.uk/species/1411666.

## Genome annotation report

The *Andrena dorsata* genome assembly (GCA_929108735.1) was annotated using the Ensembl rapid annotation pipeline ( Table 1; https://rapid.ensembl.org/Andrena_dorsata_GCA_929108735.1/Info/Index). The resulting annotation includes 24,323 transcribed mRNAs from 10,916 protein-coding and 3,489 non-coding genes.

## Methods

### Sample acquisition and nucleic acid extraction

A female *Andrena dorsata* (specimen ID Ox000751, ToLID iyAndDors1) was collected from Wytham Woods, Oxfordshire (biological vice-county Berkshire), UK (latitude 51.77, longitude –1.33) on 2020-08-04 by netting. The specimen was collected and identified by Steven Falk (independent researcher) and preserved on dry ice.

DNA was extracted at the Tree of Life laboratory, Wellcome Sanger Institute (WSI). The iyAndDors1 sample was weighed and dissected on dry ice with head tissue set aside for Hi-C sequencing. Thorax tissue was disrupted using a Nippi Powermasher fitted with a BioMasher pestle. High molecular weight (HMW) DNA was extracted using the Qiagen MagAttract HMW DNA extraction kit. Low molecular weight DNA was removed from a 20 ng aliquot of extracted DNA using the 0.8X AMpure XP purification kit prior to 10X Chromium sequencing; a minimum of 50 ng DNA was submitted for 10X sequencing. HMW DNA was sheared into an average fragment size of 12–20 kb in a Megaruptor 3 system with speed setting 30. Sheared DNA was purified by solid-phase reversible immobilisation using AMPure PB beads with a 1.8X ratio of beads to sample to remove the shorter fragments and concentrate the DNA sample. The concentration of the sheared and purified DNA was assessed using a Nanodrop spectrophotometer and Qubit Fluorometer and Qubit dsDNA High Sensitivity Assay kit. Fragment size distribution was evaluated by running the sample on the FemtoPulse system.

RNA was extracted from abdomen tissue of iyAndDors2 in the Tree of Life Laboratory at the WSI using TRIzol, according to the manufacturer’s instructions. RNA was then eluted in 50 μl RNAse-free water and its concentration assessed using a Nanodrop spectrophotometer and Qubit Fluorometer using the Qubit RNA Broad-Range (BR) Assay kit. Analysis of the integrity of the RNA was done using Agilent RNA 6000 Pico Kit and Eukaryotic Total RNA assay.

### Sequencing

Pacific Biosciences HiFi circular consensus and 10X Genomics read cloud DNA sequencing libraries were constructed according to the manufacturers’ instructions. DNA sequencing was performed by the Scientific Operations core at the WSI on Pacific Biosciences SEQUEL II (HiFi) and Illumina NovaSeq 6000 (10X) instruments. Hi-C data were also generated from head tissue of iyAndDors1 using the Arima2 kit and sequenced on the Illumina NovaSeq 6000 instrument.

### Genome assembly, curation and evaluation

Assembly was carried out with Hifiasm ( Cheng *et al.*, 2021) and haplotypic duplication was dentified and removed with purge_dups ( Guan *et al.*, 2020). One round of polishing was performed by aligning 10X Genomics read data to the assembly with Long Ranger ALIGN, calling variants with FreeBayes ( Garrison & Marth, 2012). The assembly was then scaffolded with Hi-C data ( Rao *et al.*, 2014) using YaHS ( Zhou *et al.*, 2023). The assembly was checked for contamination and corrected as described previously ( Howe *et al.*, 2021). Manual curation was performed using HiGlass ( Kerpedjiev *et al.*, 2018) and Pretext ( Harry, 2022). The mitochondrial genome was assembled using MitoHiFi ( Uliano-Silva *et al.*, 2022), which runs MitoFinder ( Allio *et al.*, 2020) or MITOS ( Bernt *et al.*, 2013) and uses these annotations to select the final mitochondrial contig and to ensure the general quality of the sequence.

A Hi-C map for the final assembly was produced using bwa-mem2 ( Vasimuddin *et al.*, 2019) in the Cooler file format ( Abdennur & Mirny, 2020). To assess the assembly metrics, the *k*-mer completeness and QV consensus quality values were calculated in Merqury ( Rhie *et al.*, 2020). This work was done using Nextflow ( Di Tommaso *et al.*, 2017) DSL2 pipelines “sanger-tol/readmapping” ( Surana *et al.*, 2023a) and “sanger-tol/genomenote” ( Surana *et al.*, 2023b). The genome was analysed within the BlobToolKit environment ( Challis *et al.*, 2020) and BUSCO scores ( Manni *et al.*, 2021; Simão *et al.*, 2015) were calculated.

Table 3 contains a list of relevant software tool versions and sources.

**Table 3.** Software tools: versions and sources.

Software tool	Version	Source
BlobToolKit	3.5.0	https://github.com/blobtoolkit/ blobtoolkit
BUSCO	5.3.2	https://gitlab.com/ezlab/busco
FreeBayes	1.3.1-17- gaa2ace8	https://github.com/freebayes/ freebayes
Hifiasm	0.15.3	https://github.com/chhylp123/ hifiasm
HiGlass	1.11.6	https://github.com/higlass/higlass
Long Ranger ALIGN	2.2.2	https://support.10xgenomics.com/genome-exome/software/pipelines/latest/advanced/other-pipelines
Merqury	MerquryFK	https://github.com/thegenemyers/ MERQURY.FK
MitoHiFi	2	https://github.com/marcelauliano/ MitoHiFi
PretextView	0.2	https://github.com/wtsi-hpag/ PretextView
purge_dups	1.2.3	https://github.com/dfguan/purge_ dups
SALSA	2.2	https://github.com/salsa-rs/salsa
sanger-tol/ genomenote	v1.0	https://github.com/sanger-tol/ genomenote
sanger-tol/ readmapping	1.1.0	https://github.com/sanger-tol/ readmapping/tree/1.1.0
YaHS	1.0	https://github.com/c-zhou/yahs

### Genome annotation

The Ensembl gene annotation system ( Aken *et al.*, 2016) was used to generate annotation for the *Andrena dorsata* assembly (GCA_929108735.1). Annotation was created primarily through alignment of transcriptomic data to the genome, with gap filling via protein-to-genome alignments of a select set of proteins from UniProt ( UniProt Consortium, 2019).

### Wellcome Sanger Institute – Legal and Governance

The materials that have contributed to this genome note have been supplied by a Darwin Tree of Life Partner. The submission of materials by a Darwin Tree of Life Partner is subject to the **‘Darwin Tree of Life Project Sampling Code of Practice’**, which can be found in full on the Darwin Tree of Life website here. By agreeing with and signing up to the Sampling Code of Practice, the Darwin Tree of Life Partner agrees they will meet the legal and ethical requirements and standards set out within this document in respect of all samples acquired for, and supplied to, the Darwin Tree of Life Project.

Further, the Wellcome Sanger Institute employs a process whereby due diligence is carried out proportionate to the nature of the materials themselves, and the circumstances under which they have been/are to be collected and provided for use. The purpose of this is to address and mitigate any potential legal and/or ethical implications of receipt and use of the materials as part of the research project, and to ensure that in doing so we align with best practice wherever possible. The overarching areas of consideration are:

•   Ethical review of provenance and sourcing of the material

•   Legality of collection, transfer and use (national and international) 

Each transfer of samples is further undertaken according to a Research Collaboration Agreement or Material Transfer Agreement entered into by the Darwin Tree of Life Partner, Genome Research Limited (operating as the Wellcome Sanger Institute), and in some circumstances other Darwin Tree of Life collaborators.

## Data Availability

European Nucleotide Archive: *Andrena dorsata* (short-fringed mining bee). Accession number PRJEB48400; https://identifiers.org/ena.embl/PRJEB48400. ( Wellcome Sanger Institute, 2022) The genome sequence is released openly for reuse. The *Andrena dorsata* genome sequencing initiative is part of the Darwin Tree of Life (DToL) project. All raw sequence data and the assembly have been deposited in INSDC databases. Raw data and assembly accession identifiers are reported in Table 1.

## Author information

Members of the University of Oxford and Wytham Woods Genome Acquisition Lab are listed here: https://doi.org/10.5281/zenodo.4789928.

Members of the Darwin Tree of Life Barcoding collective are listed here: https://doi.org/10.5281/zenodo.4893703.

Members of the Wellcome Sanger Institute Tree of Life programme are listed here: https://doi.org/10.5281/zenodo.4783585.

Members of Wellcome Sanger Institute Scientific Operations: DNA Pipelines collective are listed here: https://doi.org/10.5281/zenodo.4790455.

Members of the Tree of Life Core Informatics collective are listed here: https://doi.org/10.5281/zenodo.5013541.

Members of the Darwin Tree of Life Consortium are listed here: https://doi.org/10.5281/zenodo.4783558.
