# Supplementary material for: The genome sequence of the short-fringed mining bee, Andrena dorsata (Kirby, 1802)
Source: Wellcome Open Res. 2023 Aug 30;8:373. [Version 1] doi: 10.12688/wellcomeopenres.19756.1 (PMC11305455; doi:10.12688/wellcomeopenres.19756.1)
Supplement: Peer review history

# Reviewer report: Reviewer response for version 1

**DOI:** 10.21956/wellcomeopenres.21882.r88320

**Published:** 2024-08-06

**Authors:** Christoph Bleidorn

**Affiliations:** 1 Department of Animal Evolution and Biodiversity, University of Göttingen, Göttingen, Germany

The manuscript "The genome sequence of the short-fringed mining bee, *Andrena dorsata *(Kirby, 1802)" by Falk and Mulley reports a high-quality chromosome-resolved genome sequence of the short-fringed mining bee (Andrena dorsata). This rather common bee species is bivoltine and is polylectic regarding its pollen preference (this fact could be mentioned). There is a closely related taxon called *Andrena propinqua*, which sometimes has been regarded as synonymous with *A. dorsata*, but UCE data support its status as an own species (Gueuning, Morgan et al. (2020 ^1^ )). This confusing taxonomy might also contribute to the classification as being "data deficient" as mentioned by the authors. I would suggest to mention this taxonomic background.

 The reported genome matches the very high standard of DTOL genomes. The methods are state-of-the-art and the genome assembly resulted in a chromosome-scale resolution. The material and methods provide sufficient information regarding the data generation and analysis. While the main focus of the article is the description of a new genome, I would nevertheless suggest to include some more information of chromosome numbers in Andrena species in the introduction. The here presented case with only 3 chromosomes seems remarkable, given that most of the so-far sequenced Andrena genomes report 7 chromosomes (see DTOL genomes). The here provided genome sequence will be a useful resource for future studies on chromosome evolution in andrenin bees.

Are sufficient details of methods and materials provided to allow replication by others?

Yes

Is the rationale for creating the dataset(s) clearly described?

Yes

Are the datasets clearly presented in a useable and accessible format?

Yes

Are the protocols appropriate and is the work technically sound?

Yes

Reviewer Expertise:

Molecular phylogeny and evolution

I confirm that I have read this submission and believe that I have an appropriate level of expertise to confirm that it is of an acceptable scientific standard.

## References

[1] : Ultraconserved yet informative for species delimitation: Ultraconserved elements resolve long-standing systematic enigma in Central European bees. *Mol Ecol* .2020;29(21) : 10.1111/mec.15629 4203-4220 10.1111/mec.15629 32916006

# Reviewer report: Reviewer response for version 1

**DOI:** 10.21956/wellcomeopenres.21882.r88318

**Published:** 2024-07-22

**Authors:** Manee M Manee

**Affiliations:** 1 King Abdulaziz City for Science and Technology, Riyadh, Saudi Arabia

The study presents a comprehensive genome assembly of the short-fringed mining bee, *Andrena dorsata*. The assembly has a high consensus quality value (QV) of 53.5 and a k-mer completeness of 99.99%. BUSCO analysis indicates 96.8% completeness for hymenoptera_odb10 reference genes. I am pleased with the current form of the study.

Are sufficient details of methods and materials provided to allow replication by others?

Yes

Is the rationale for creating the dataset(s) clearly described?

Yes

Are the datasets clearly presented in a useable and accessible format?

Yes

Are the protocols appropriate and is the work technically sound?

Yes

Reviewer Expertise:

bioinformatics, genomics, and evolutionary biology

I confirm that I have read this submission and believe that I have an appropriate level of expertise to confirm that it is of an acceptable scientific standard.
